# Pressure Monitoring Cell for Constrained Battery Electrodes

**DOI:** 10.3390/s18113808

**Published:** 2018-11-06

**Authors:** Jan Patrick Singer, Christian Sämann, Tobias Gössl, Kai Peter Birke

**Affiliations:** 1 Electrical Energy Storage Systems, Institute for Photovoltaics, University of Stuttgart, 70569 Stuttgart, Germany; tobias.goessl@googlemail.com (T.G.); peter.birke@ipv.uni-stuttgart.de (K.P.B.); 2 Sensor Technology, Institute for Photovoltaics, University of Stuttgart, 70569 Stuttgart, Germany; christian.saemann@ipv.uni-stuttgart.de

**Keywords:** battery test cell, cell pressure, volume change, constrained batteries, pressure measurement, electrode testing

## Abstract

Testing of improved battery components and new electrochemical energy storage materials in a coin cell format as a test cell is becoming the state of the art. The pressure on the electrode surfaces inside an electrochemical cell is one of the important parameters for high ionic/electronic conductivity and the cyclic lifetime. A self-designed pressure monitoring cell allows both applying an adjustable pressure and monitoring the state of charge-dependent cell pressure during cycling. The load cell shows a reciprocal behavior of the temperature sensitivity dependent on the ambient temperature and requires constant temperature conditions while monitoring the cell pressure. Further, dependent on the initial cell pressure, the relaxation time of the assembled pressure monitoring cell must be considered. The present paper describes the setup, the influence of the environment temperature and the mechanical relaxation of the pressure monitoring cell. The first cycling results, using an NCM/graphite coin cell, demonstrate the functionality of the pressure monitoring cell measuring the cell’s pressure as a function of the C-rate.

## 1. Introduction

Currently, due to increasing performance requirements mainly driven by the automotive sector, plenty of research on battery electrodes has been published in the literature [1,2,3,4,5,6]. The main requirements are increasing volumetric and gravimetric energy density, as well as power density, higher cyclic lifetime and replacing toxic components with environmentally-friendly compounds. Therefore, two trends are identifiable: first, improvement of existing and usable materials and, second, finding and making new materials that are able to be used as electrochemical energy storage materials, as well as finding compatible electrolytes. Thereby, potential storage materials were, for example, assembled as an electrode in the format of a coin cell and were cycled in a test coin cell [7,8,9]. Most of the electrochemical energy storage materials undergo volume changes during the insertion/extraction of the charge transfer species [4,10]. The volume changes cause accelerated aging of the electrode due to particle cracking [11] and are an indicator of the State of Health (SoH) [12]. In order to improve the electrode materials, the measurement and control of the volume expansions of the electrodes are mandatory. Dilatometer cells with displacement sensors are commercially available and allow the measurement of the expansion in the nanometer range [13]. In applications, battery cells are usually constrained with a defined force [14]. Constraining cells enables a higher cyclic lifetime and a good ionic conductivity within the cell [15].

This paper presents a self-constructed pressure monitoring cell. The test cell allows the constraining of coin cell electrodes with a diameter of d= 18 mm within a defined pressure range $0\leq p_{\mathrm{cell}}\leq 2$ MPa. Further, we monitor the cell pressure during cycling of the cell and are able to measure the increasing cell pressure as a consequence of increasing volume change due to interface formation and aging processes [12,16].

## 2. Method and Working Principle

### 2.1. Test Cell Setup

**Figure 1:**
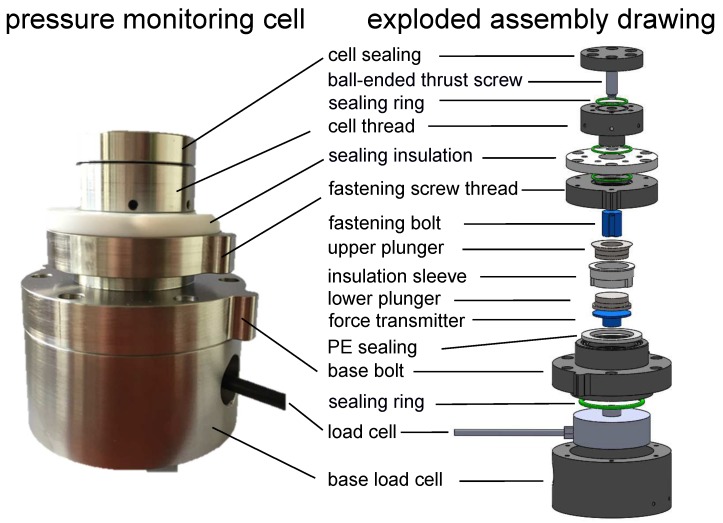
Picture and exploded assembly drawing of the pressure monitoring cell with the corresponding labeled components. The load cell is placed in the bottom part of the monitoring cell and connected to the inner part of the cell setup via a force transmitter. The inner part of the pressure monitoring cell consists of EL-Cell^®^ GmbH, Germany, components. A ball-ended thrust screw in the upper part of the monitoring cell enables the adjustment of the initial pressure. Several sealing levels ensure the leakproofness.

For battery electrodes, as well as the electrolyte, it is mandatory to operate within an oxygen- and humidity-free and, in special cases, even a nitrogen- and carbon dioxide-free atmosphere. The pressure monitoring cell is hermetically sealed, which prevents contamination by oxygen, humidity and other potential gases. The assembling of the cell takes place within an argon atmosphere. As the electrode/separator/electrode setup, we use the commercial products of EL-Cell^®^ GmbH, Germany, consisting of a lower plunger, upper plunger and an insulation sleeve; see Figure 1. The force is transmitted to the load cell, which is placed in the base load cell and sealed with the base bolt. A sealing ring ensures the seal tightness. With a fastening bolt, the adjusted pressure is transmitted to the upper plunger. On the top of the fastening screw thread, which seals the upper part of the cell, a sealing insulation is mounted. This sealing insulation prevents an electric short circuit within the cell. A ball-ended thrust screw enables the adjustment of the desired cell pressure after assembling the cell. The cell sealing guarantees the leakproofness of the adjustment unit. This kind of construction ensures a frictionless force transmission to the active materials and the load cell. All inner components of the cell are mechanically uncoupled to the housing and sealing system. Thus, the load cell measures the applied force by tightening the ball-ended thrust screw to the electrodes and the change of the force during cycling.

### 2.2. Load Cell and Data Acquisition

**Figure 2:**
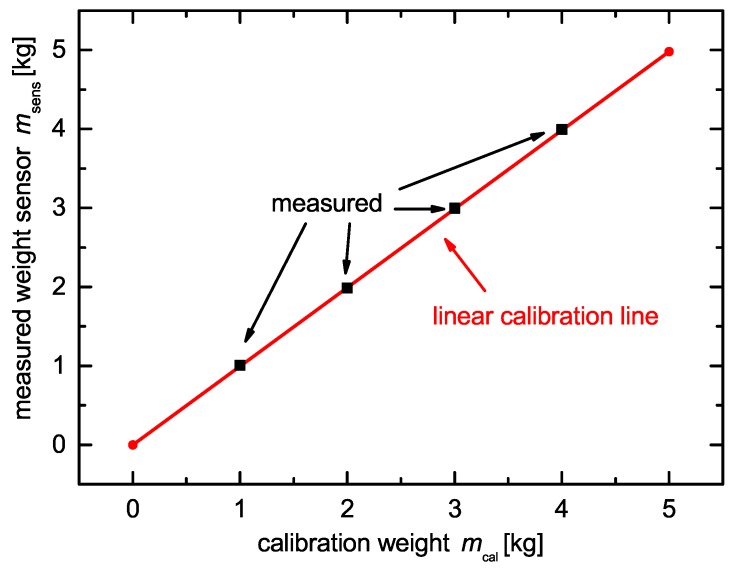
Linear calibration line of the load cell and measured weights using the calibration line. The calibration line is used as a look up table by the Arduino software.

The load cell CM−0.5 kN by X-Senors GmbH, Germany, measures the force change relative to an initial force as a consequence of the volume change during insertion/extraction of the charge transfer species in the active materials. The nominal force of the load cell is $F_{\mathrm{nom}}=$ 0.5 kN within a tolerance of the output signal s≤±0.2% Full Scale (F.S.), a linearity *l* and hysteresis *h* of l=h≤±0.2% F.S. A 24-bit analog-digital converter HX711 by Avia Semiconductors converts the analog voltage signal of the load cell, while an Arduino Mega 2560 calculates and stores the pressure $p_{\mathrm{cell}}$ values. In order to calibrate the load cell, we use precision calibration weights. Figure 2 shows the calibration result of the load cell with calibration weights. One can see that the linear calibration line is confirmed by measuring calibration weights. The obtained calibration factor of the calibration line is used by the Arduino software for the calculation of the weight. The resolution, as well as the accuracy of the load cell equates to the measurement precision of the pressure monitoring cell.

## 3. Results and Discussion

### 3.1. Temperature Sensitivity

**Figure 3:**
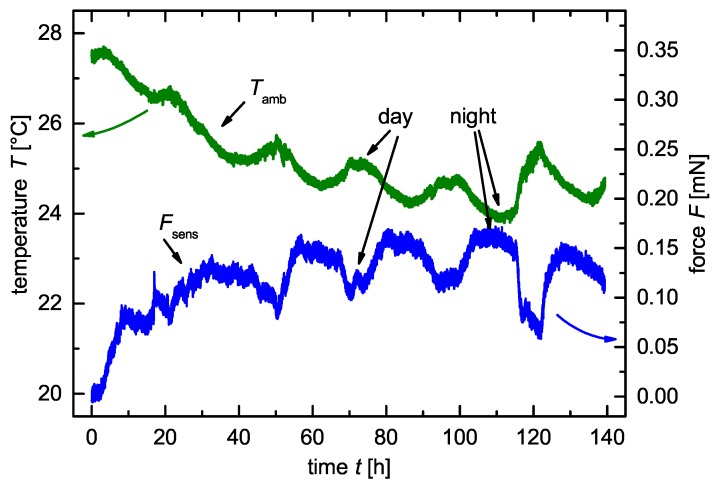
Fluctuating ambient temperature $T_{\mathrm{amb}}$ in the laboratory and the inverse proportional measured force *F* over a time period of Δt=140 h.

For the temperature validation of the test cell, we rested the cell unloaded at ambient temperature in our laboratory for t=140 h. Figure 3 shows the force *F* and the ambient temperature $T_{\mathrm{amb}}$ independent of the time *t*. The force *F* correlates inversely proportional to the ambient temperature $T_{\mathrm{amb}}$ in a day and night cycle.

As the sensor signal of the load cell depends on the ambient temperature $T_{\mathrm{amb}}$, it was necessary to cycle the pressure monitoring cell at a constant temperature. According to this observation, we placed the cell within a climate chamber at a defined temperature with a variance of ΔT±0.1 K.

### 3.2. Mechanical Relaxation

To determine the mechanical relaxation behavior of the test cell, we applied several initial pressures on the cell $p_{\mathrm{init}}=$ 0.1 MPa, 0.25 MPa, 0.5 MPa, 0.75 MPa and 1 MPa. Therefore, we assembled the cell according to Figure 1 without any electrodes or a separator. Afterwards, we applied the initial pressure and placed the cell within the climate chamber with an ambient temperature T=25${}^{\circ }C$ for t>15 h.

**Figure 4:**
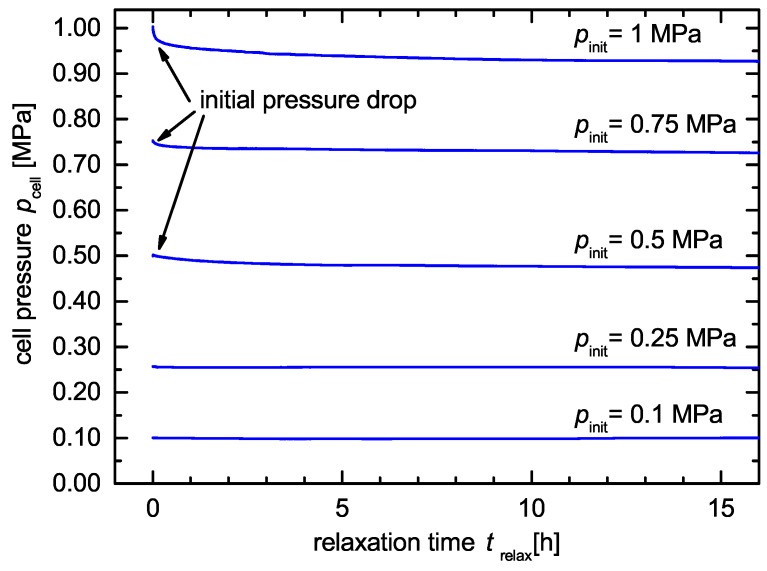
Mechanical relaxation of the pressure monitoring cell for $p_{\mathrm{init}}=$ 0.1 MPa, 0.25 MPa, 0.5 MPa, 0.75 MPa and 1 MPa. An increasing initial pressure leads to an increasing relaxation time $t_{\mathrm{relax}}$ and a higher initial pressure drop.

For $p_{\mathrm{init}}=$ 0.1 MPa and 0.25 MPa, we could not observe a mechanical relaxation of the cell, see Figure 4. The small change in the measured cell pressure curve $p_{\mathrm{cell}}$ can be explained by the tolerance of the climate chamber. During the alternation of day and night, the climate chamber’s temperature changed because of the changing room temperature within the variance of ΔT±0.1 K. For $p_{\mathrm{init}}>$ 0.5 MPa, we observed an initial pressure drop of $p_{\mathrm{cell}}$ followed by a relaxation time. A re-adjustment of the initial pressure $p_{\mathrm{init}}$ should be performed therefore after the relaxation time $t_{\mathrm{relax}}$.

### 3.3. Cell Pressure

**Figure 5:**
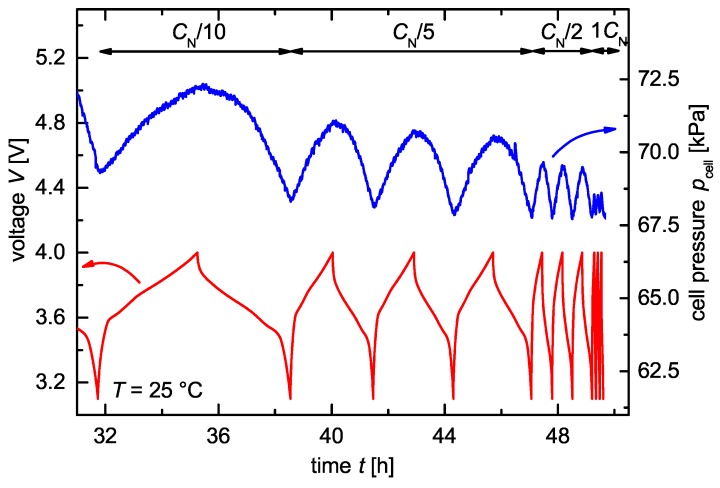
Pressure-time and voltage-time curves of a constrained NCM/graphite coin cell at several C-rates using a pressure monitoring cell for constrained battery electrodes. The cell pressure $p_{\mathrm{cell}}$ correlates with the voltage *V* of the electrochemical cell. The pressure difference during cycling decreases with increasing current.

Figure 5 shows the time-dependent voltage and pressure behavior of an NCM/graphite cell during cycling with a current of $I=C_{N}/10$, $I=C_{N}/5$, $I=C_{N}/2$ and $I=1C_{N}$ at a temperature T=25${}^{\circ }C$. The nominal capacity of the graphite anode $C_{N},A=$ 9.7 mAh ± 5% and of the NCM cathode $C_{N},C=$ 9 mAh ± 5% was not reached, which is visible if looking at the time scale. Both electrodes were produced by Custom Cells Itzehoe GmbH, Germany. A borosilicate-glass fiber separator GF/A by Whatman^©^, United Kingdom, isolated the electrodes electrically, while a LP40 electrolyte by BASF AG, Germany, ensured the ionic conductivity.

During the charge process, the volume of the anode expanded up to $\Delta V_{a}/V_{0}=12.8$% [17], and the cathode shrank down to $\Delta V_{c}/V_{0}=2.44$% [18]. The resulting overall volume change $\Delta V_{\mathrm{tot}}$ of a cell is the difference between the expanding anode and the shrinking cathode $\Delta V_{\mathrm{tot}}/V_{0}=\Delta V_{a}/V_{0}-\Delta V_{c}/V_{0}=10.36$%. In a constrained test cell, the cell’s volume could not expand and resulted in an increasing pressure during cycling. The pressure $p_{\mathrm{cell}}$ in Figure 5 increased simultaneously with the voltage *V* and reached its maximum at the end of charge voltage. The other way around, the lowest cell pressure was measured at the end of the discharge voltage. Further, we can identify a decreasing absolute pressure with an increasing C-rate. This C-rate-dependent pressure behavior corresponds to the literature [19,20,21]. The reason for the C-rate-dependent pressure amplitude is still under discussion in the community. However, the main reasons might be the thermal expansion [20] and missing mechanical relaxation because of a non-equilibrium state as a consequence of high kinetics [19] due to high C-rates. Further, changes in phase transition processes during the insertion/extraction of charge carriers in the host material [21] are under discussion. The measurement results in Figure 5 conclude an operating and leakproof test cell, a sufficient pressure resolution and a force transmission within the cell.

## 4. Conclusions

In this study, we demonstrate a concept to setup and monitor the cell pressure of constrained battery electrode in a novel battery test cell. Measurement results of the time-dependent voltage *V* and pressure curve $p_{\mathrm{cell}}$ confirm the functionality of the pressure monitoring test cell. We are also able to detect the C-rate-dependent pressure change during cycling, which enables a wide range of investigations. In future work, we will determine the influence of the external cell pressure on the formation of the electrode’s surface layers. Further, the investigation of an optimum pressure for different electrode materials will be targeted to reach a maximum of the cycling lifetime. Finally, detailed investigations for a better understanding of the C-rate-dependent pressure amplitude are scheduled.
